# Change in Long Non-Coding RNA Expression Profile Related to the Antagonistic Effect of *Clostridium perfringens* Type C on Piglet Spleen

**DOI:** 10.3390/cimb45030149

**Published:** 2023-03-09

**Authors:** Zunqiang Yan, Pengfei Wang, Qiaoli Yang, Xiaoli Gao, Shuangbao Gun, Xiaoyu Huang

**Affiliations:** College of Animal Science and Technology, Gansu Agricultural University, Lanzhou 730070, China

**Keywords:** piglet, *Clostridium perfringens* type C, spleen, RNA-Seq, diarrhea

## Abstract

**Simple Summary:**

*Clostridium perfringens* (*C. perfringens*) type C is a spore-forming pathogenic bacterium characterized by the secretion of fatal toxins, which are absorbed into the body, causing diarrhea. Diarrhea has already brought about tremendous economic losses in pig farms worldwide. However, the understanding of lncRNAs’ regulatory mechanisms of the spleen in piglets challenged by *C. perfringens* type C is still limited. This paper aimed to identify antagonistic lncRNAs associated with the spleen in piglets challenged by *C. perfringens* type C. The study found that four lncRNAs are involved in immune-/inflammation-related pathways to regulate cytokine genes against *C. perfringens* type C infection.

**Abstract:**

LncRNAs play important roles in resisting bacterial infection via host immune and inflammation responses. *Clostridium perfringens* (*C. perfringens*) type C is one of the main bacteria causing piglet diarrhea diseases, leading to major economic losses in the pig industry worldwide. In our previous studies, piglets resistant (SR) and susceptible (SS) to *C. perfringens* type C were identified based on differences in host immune capacity and total diarrhea scores. In this paper, the RNA-Seq data of the spleen were comprehensively reanalyzed to investigate antagonistic lncRNAs. Thus, 14 lncRNAs and 89 mRNAs were differentially expressed (DE) between the SR and SS groups compared to the control (SC) group. GO term enrichment, KEGG pathway enrichment and lncRNA-mRNA interactions were analyzed to identify four key lncRNA targeted genes via MAPK and NF-κB pathways to regulate cytokine genes (such as TNF-α and IL-6) against *C. perfringens* type C infection. The RT-qPCR results for six selected DE lncRNAs and mRNAs are consistent with the RNA-Seq data. This study analyzed the expression profiling of lncRNAs in the spleen of antagonistic and sensitive piglets and found four key lncRNAs against *C. perfringens* type C infection. The identification of antagonistic lncRNAs can facilitate investigations into the molecular mechanisms underlying resistance to diarrhea in piglets.

## 1. Introduction

*Clostridium perfringens* (*C. perfringens*) type C is one of the most devastating pathogens related to diarrhea, necrotizing enteritis and struck in animals [1]. Recently, *C. perfringens* type C-induced diarrhea characterized by a high morbidity and mortality has frequently occurred at large-scale pig farms, leading to huge economic losses around the world [2,3]. Pigs, especially piglets, are infected with this bacterium mainly via the oral digestive tract. Then, an increase in the number of *C. perfringens* type C bacteria in the small intestine can secrete fatal toxins (at least α and β), which impair tight junction integrity and damage the passages of water and solutes, causing diarrhea [4,5]. Additionally, fatal toxins are usually absorbed by the small intestine and then transported to terminal organs (such as the spleen, liver and brain), leading to host tissue injury and organ damage [3,6,7,8,9]. The traditional method of preventing and controlling this bacterium is antibiotic therapy, although this approach has some disadvantages (including bacterial resistance to many antibiotics and antibiotic residues in pork) [2,5]. Under pressure from the public, the United States and the European Union have taken some measures to ban the application of some medical antibiotics for disease prevention and growth promotion [10,11]. Therefore, there is an urgent need to find a new method for preventing and controlling diarrhea caused by *C. perfringens* type C infection.

The difference in host resistance to pathogens (such as bacteria) is associated with host immunity, pathogen exposure and the interaction of host defense and pathogen virulence [12,13]. Pigs with a G to A mutation at locus M307 of *FUT1* can resist Enterotoxigenic *Escherichia coli* F18 infection [14]. Moreover, White Leghorn chicken line 6.3 is resistant to Marek’s disease, and line 7.2 is susceptible to Marek’s disease [15]. Additionally, animals with a low immunity are more sensitive to pathogens than those with a strong immunity [16,17,18]. These results suggest that the identification of candidate molecules with resistant pathogens is beneficial for the breeding of new lines for preventing and controlling infectious diseases. At present, the understanding of the molecular mechanism of piglet resistance to *C. perfringens* type C infection is still limited. Thus, elucidating the underlying molecular regulation of different levels of resistance in piglets challenged by *C. perfringens* type C will be an effective approach to artificially selecting resistant piglets for controlling diarrhea prevalence and to find some molecular markers of *C. perfringens* type C resistance.

Long non-coding RNA (lncRNA) is a class of non-coding RNAs with more than 200 nucleotides and present in many species, such as pigs [19], zebrafish [20] and humans [21]. Recently, lncRNAs are receiving more attention because of their important regulatory roles in biological processes, such as normal development, metabolic diseases, cardiovascular diseases and tumor formation [22,23]. Furthermore, lncRNAs also play important roles in autoimmune diseases (such as autoimmune hepatitis [24] and systemic lupus erythematosus [25]) and infectious diseases (including diarrhea [26], malaria [27] and African trypanosomes [28]). However, lncRNAs and their target genes related to *C. perfringens* type C resistance in piglets are poorly understood. The use of RNA-Seq to identify lncRNAs against *C. perfringens* type C infection in pigs is the basis for revealing the molecular mechanism of disease resistance and, thus, for discovering candidate genes related to disease resistance traits.

The spleen is the main immune organ, and it plays important roles in filtering blood-borne pathogens and antigens to protect the host against various infectious diseases and pathogen infections [29,30,31]. Herein, the spleen is regarded as the ideal organ model for exploring host resistance and susceptibility to pathogenic challenges. Studies of disease resistance have been performed in sheep spleen [32,33], chicken spleen [15,34] and pig spleen [35]. Additionally, differentially expressed lncRNAs and mRNAs in two spleen phenotypes, antagonistic or sensitive to *C. perfringens* type C, were identified using RNA-Seq in our previous study [36]. However, our previous research only focused on identifying molecules associated with resistance and susceptibility to *C. perfringens* type C, and we did not further investigate how these molecules function in the process of *C. perfringens* type C infection. In a continuation of our previous research, the aim of this study was to comprehensively identify and reanalyze the dysregulated lncRNAs and mRNAs in the spleen of resistant and sensitive piglets. Our study provides new insights into piglet antagonism to *C. perfringens* type C in terms of lncRNAs, which also contributes to formulating a breeding strategy against *C. perfringens* type C infection.

## 2. Materials and Methods

### 2.1. Experimental Design and Sample Collection

The experimental piglets were purchased from the nucleus herd in Dingxi city, Gansu province. We randomly selected a total of 30 seven-day-old piglets displaying normal growth and approximately similar body weight. Additionally, the piglets were not infected with *Escherichia coli*, *Salmonella* or *C. perfringens*, as tested using ELISA kits (Jiancheng Bioengineering Institute, Nanjing, Jiangsu, China). Among these piglets, 5 piglets were selected to form the control group (SC). The other 25 piglets were challenged by a *C. perfringens* type C strain (CVCC 2032), and the top 5 and bottom 5 piglets according to total diarrhea scores were considered the susceptible group (SS) and the resistant group (SR) by using a previously described method [36,37].

The spleen and other tissues from the piglets in the SC, SR and SS groups were collected. Then, these tissues were frozen in liquid nitrogen and stored at −80 °C until used for RNA isolation. Moreover, the spleen was obtained and stored at −80 °C for detecting cytokine expression levels using RT-qPCR and concentration using ELISA.

### 2.2. Total RNA Isolation

The total RNA was extracted from each individual sample using the TRIzol reagent (Invitrogen, Carlsbad, CA, USA). In addition, the purity and quantity of the total RNA were determined using a Nanodrop instrument (Implen, Westlake Village, CA, USA). The integrity of the spleen total RNA was measured using a Bioanalyzer 2100 system (Agilent Technologies, Santa Clara, CA, USA).

### 2.3. Library Preparation for lncRNA Sequencing

A total of 3 μg of spleen total RNA was used to construct lncRNA sequencing libraries by utilizing a previous method [36]. Furthermore, the lncRNA sequencing libraries were sequenced on an Illumina Hiseq 4000 platform, and 150 bp paired-end reads were obtained at the Novogene Bioinformatics Institute (Beijing, China).

### 2.4. Quality Control and Mapping

The raw reads (raw data) were first processed using in-house Perl scripts. In this step, clean reads (clean data) were generated by removing reads that contained adapters or over 10% of ploy-N, or low-quality reads (>50% of bases whose Phred scores were <5%) based on the raw data. At the same time, the Phred score (Q20, Q30) and GC content of the clean reads were assessed. The clean reads were mapped to the pig reference genome with Tophat (2.0.9 version) [38].

### 2.5. Transcriptome Assembly and Expression Level Quantification

The mapped reads of each individual sample were assembled using Cufflinks (2.1.1 version) [38] and Scripture (beta2 version) [39]. Cuffdiff (2.1.1 version) [40] was used to evaluate the lncRNA and mRNA expression levels by Fragments Per Kilobase Million (FPKM). For biological replicates, the lncRNAs and mRNAs with a *P*-adjust < 0.05 were described as differentially expressed (DE) among three group comparisons (SR vs. SC, SR vs. SS and SS vs. SC).

### 2.6. Coding Potential Analysis and Target Gene Prediction

To achieve highly reliable novel lncRNAs, previously stringent filtering criteria were used [36]. In addition, protein-coding genes 100 k downstream and upstream of the lncRNAs were considered the *cis* target genes. The *trans* target genes of the lncRNAs were obtained by examining the expression levels of the lncRNAs and mRNAs with custom scripts (Pearson correlation coefficient ≥0.95).

### 2.7. Enrichment Analysis of GO and KEGG

Gene ontology (GO) enrichment analyses of DE lncRNA target genes and DE mRNAs were implemented using the GOseq R package (1.50.0 version) [41]. KEGG pathway analyses of DE lncRNA target genes and DE mRNAs were performed using KOBAS software (3.0 version) [42].

### 2.8. Heat Map Construction and lncRNA Secondary Structure Prediction

A hierarchical heat map analysis was performed using OmicShare tools, a free online platform for data analyses (http://www.omicshare.com/tools (accessed on 8 November 2022)). The prediction of the secondary structure of the lncRNAs was conducted based on the free energy using the RNAFold web server online tool (http://rna.tbi.univie.ac.at/cgi-bin/RNAWebSuite/RNAfold.cgi (accessed on 11 December 2022)).

### 2.9. RT-qPCR Assay and ELISA Detection

The total RNA of the spleen tissues used for RNA-Seq were reverse-transcribed into cDNA using a PrimeScript™ RT Reagent kit (Takara, Dalian, China). The primers were designed in Primer3, and primer specificity was assessed using Primer-BLAST (Table S1). An RT-qPCR assay was performed in a reaction system containing 7.5 µL of RNase free ddH_2_O, 1 µL of cDNA, 1 µL of reverse primer, 1 µL of forward primer and 9.5 µL of the SYBR^®^ Green PCR Master Mix (Takara, Dalian, China) using a LightCycler 480II instrument (Roche, Basel, Switzerland). The thermal cycler conditions included an initial pre-denaturation at 95 °C for 3 min and 40 cycles at 95 °C for 15 s, 58 ± 1 °C for 15 s and 72 °C for 20 s. LncRNA and mRNA expressions were quantified relative to *β-actin* expression using the 2^−∆∆Ct^ method [43]. All ELISA processes were conducted according to the manufacturer’s instructions. The concentrations of cytokines (including TNF-α, IFN-γ, IFN-α, IL-6 and IL-8) in the spleen tissue were determined using an ELISA kit (Kete Biotech, Yancheng, Jiangsu, China). The RT-qPCR and ELISA data are presented as Mean ± SE. A one-way ANOVA was performed to calculate statistical significance followed by Duncan’s test using SPSS (25.0 version).

## 3. Results

### 3.1. Analyses of Differentially Expressed lncRNAs

**Figure 1:**
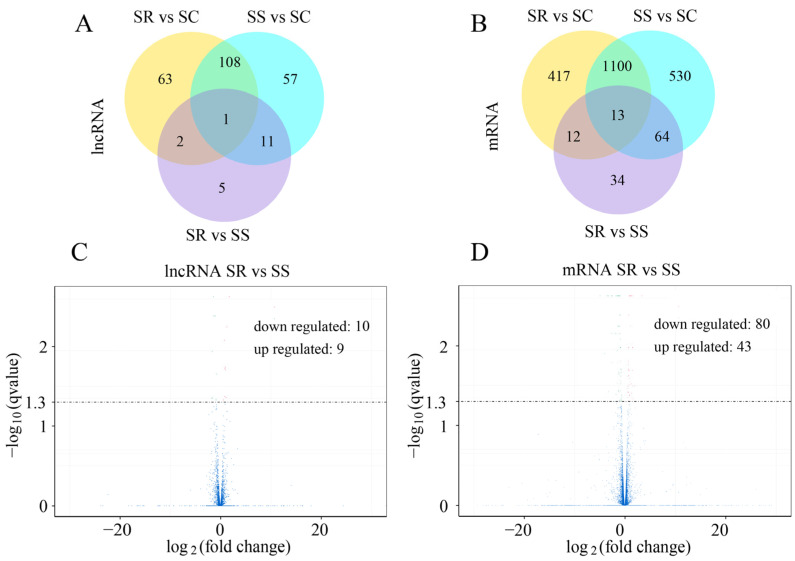
Analysis of DE lncRNAs and mRNAs in the spleen. Venn diagram showing DE lncRNAs (**A**) and mRNAs (**B**) among SR vs. SC, SS vs. SC and SR vs. SC groups. Volcano plot presenting DE lncRNAs (**C**) and mRNAs (**D**) between SR and SC groups.

**Figure 2:**
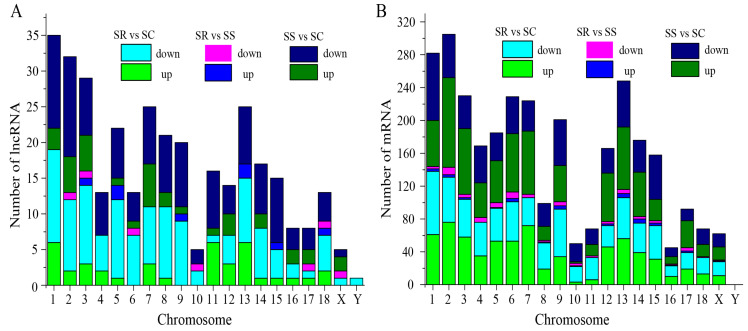
Distributions of DE lncRNAs and mRNAs in pig chromosome. The *x*-axis indicates different chromosomes, and the *y*-axis indicates the number of lncRNAs or mRNAs.

To identify the lncRNAs and mRNAs in the *C. perfringens* type C-challenged piglets in the SR, SS and SC groups, RNA libraries of the spleen samples were constructed and sequenced. The results indicate that 177 lncRNAs (including 45 down-regulated and 132 up-regulated) and 1707 mRNAs (including 919 down-regulated and 788 up-regulated) were significantly dysregulated between the SS and SC groups (Figure 1A,B and Table S2). A total of 174 lncRNAs and 1542 mRNAs between the SR and SC groups were differentially expressed (Figure 1A,B, and Table S3). A total of 19 lncRNAs (including 10 down-regulated and 9 up-regulated) and 123 mRNAs (including 80 down-regulated and 43 up-regulated) were identified between the SR and SS groups (Figure 1C,D). In addition, 1 lncRNA and 13 mRNAs were significantly expressed among the three SR, SS and SC groups (Figure 1A,B). According to the visible lncRNA and mRNA levels in the SR, SS and SC groups, the DE lncRNAs were distributed across all chromosomes. Chromosomes 1, 2, 3, 7 and 13 displayed more DE lncRNAs (Figure 2A). The distribution densities of the DE mRNAs were different. Most of the DE mRNAs were distributed among chromosomes 1, 2 and 13. However, there were no DE mRNAs in chromosome Y (Figure 2B). Compared to the SC group, a total of 14 lncRNAs and 89 mRNAs were found to be differentially expressed between the SR and SS groups (Figure 1A,B). Table 1 shows the detailed information of these molecules, which were used as potential resources for identifying the lncRNAs related to the antagonistic effects of *C. perfringens* type C on piglet spleen.

**Figure 3:**
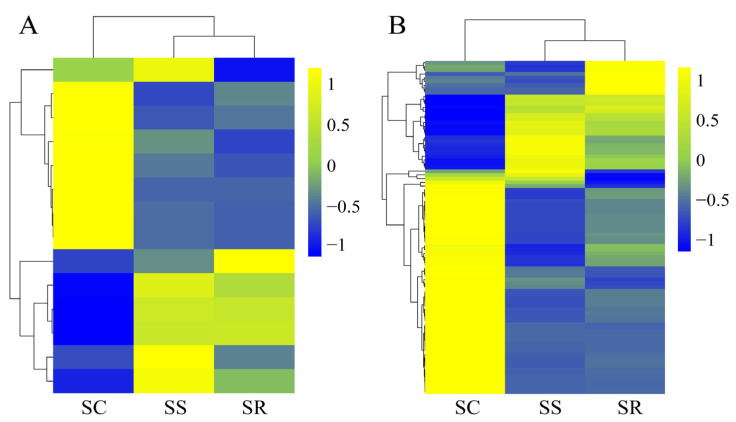
Hierarchical heat map showing the expression values for lncRNAs (**A**) and mRNAs (**B**).

To investigate the expression patterns among the three SR, SS and SC groups, we used the 14 DE lncRNAs and 89 DE mRNAs to generate a hierarchical heat map. The heat map of the DE lncRNAs and mRNAs in the spleen of the three groups revealed that the SS and SR groups were clustered together because of their similar expression profiles (Figure 3A,B).

### 3.2. Characterization of lncRNA Subtypes

**Figure 4:**
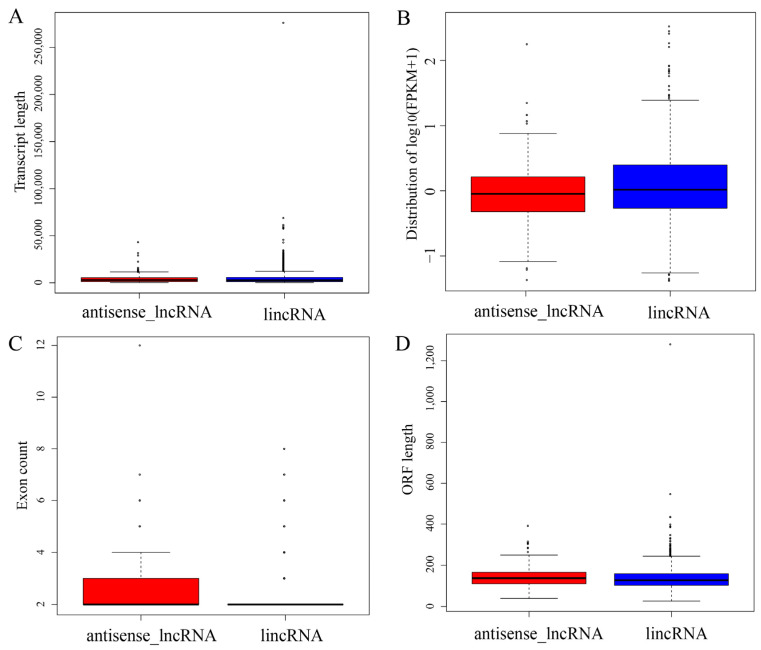
Genomic features of the different subtypes of lncRNAs. (**A**)Transcript length, (**B**) expression level, (**C**) exon count and (**D**) ORF length of different subtypes of lncRNAs were compared using the Kolmogorov–Smirnov test. Each region of the box plot indicates the maximum, upper quartile, median, lower quartile and minimum from top to bottom.

Previous studies have noted major differences in gene structures and expression levels among two subtypes of lncRNAs. Thus, the transcript length, expression level, exon count and ORF length among the different subtypes of lncRNAs were analyzed. The lengths of lincRNAs were greater than those of antisense lncRNAs, with a mean length of 4.778 kb vs. 4.353 kb, respectively. There were no significant differences in length (*p* = 0.9118, Figure 4A). In particular, the lincRNAs showed a higher expression level than the antisense lncRNAs (*p* = 0.0212, Figure 4B). Clear differences in the exon count (*p* = 0.0003, Figure 4C) and ORF length (*p* = 0.0051, Figure 4D) were observed between the two lncRNA subtypes.

### 3.3. Functional Analyses of C. perfringens Type C-Responsive lncRNAs and mRNAs

**Figure 5:**
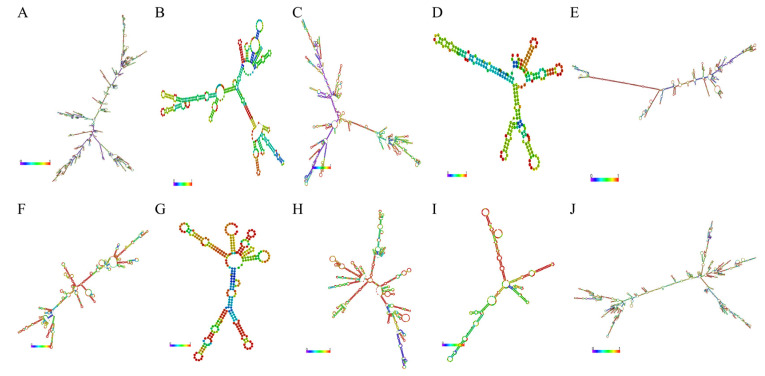
The secondary structures of 10 lncRNAs. (**A**) LNC_001595; (**B**) ALDBSSCT0000003048; (**C**) LNC_000191; (**D**) ALDBSSCT0000009442; (**E**) LNC_001065; (**F**) ALDBSSCT0000006918; (**G**) LNC_000263; (**H**) LNC_002009; (**I**) ALDBSSCT0000007366; (**J**) LNC_000042.

The secondary structures of the lncRNAs were predicted, which was helpful in recognizing the functions of these lncRNAs. A total of 10 lncRNAs had secondary structures, which mainly included a hairpin loop, a stem loop, an inner ring, a bulge ring and a multi-branch loop (Figure 5). Nevertheless, four lncRNAs (namely, *LNC_001987*, *LNC_001097*, *LNC_001253* and *LNC_001985*) had no secondary structures because of their excessive lengths.

**Figure 6:**
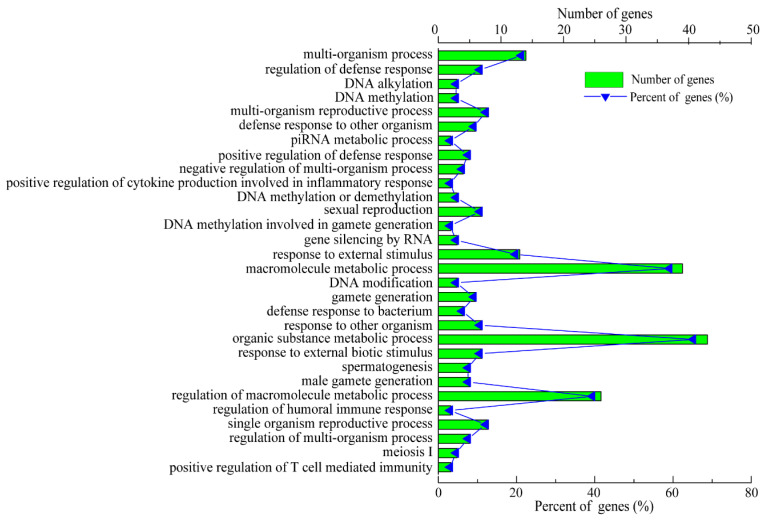
GO term analysis of the biological functions of the identified lncRNA target genes.

**Figure 7:**
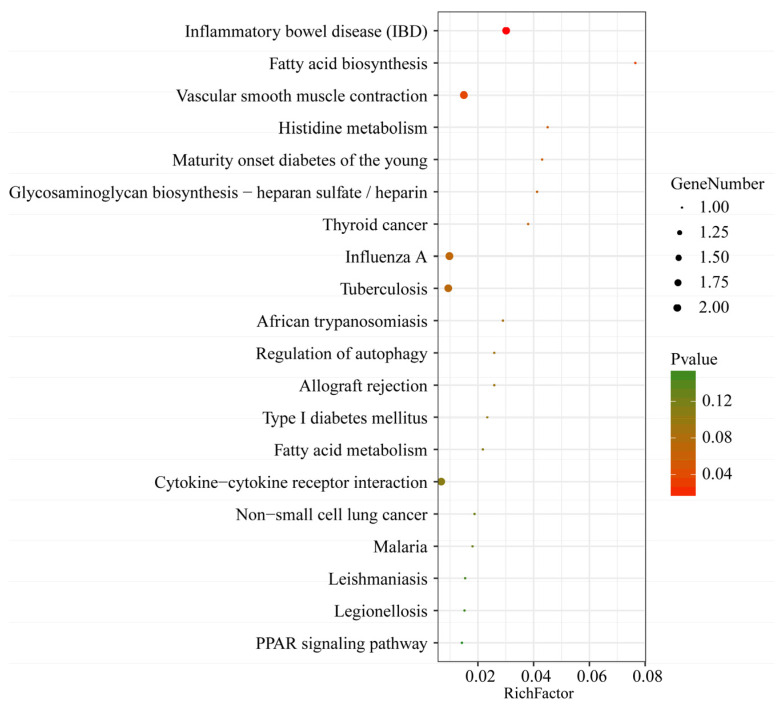
The top 20 KEGG pathways of lncRNA target genes. The *x*-axis indicates the gene ratio, and the *y*-axis indicates the name of the KEGG pathway. The size of the dot indicates the number of target genes, and the color of the dot indicates different *p* values.

To further investigate the functions of the lncRNAs, the potential target genes of the 14 identified lncRNAs in *cis* (co-location) and *trans* (co-expression) were predicted. The prediction results show that a total of 17 interaction relationships were established in *cis* between 8 lncRNAs and 17 protein-coding genes in the pig genome (Table S4). In addition, these results indicate that 4 lncRNAs corresponded to 93 protein-coding genes within a range of 100 kb *in trans* (Table S4). However, the target genes of *LNC_001595*, *LNC_000191*, *LNC_001065* and *LNC_000042* were not predicted because of a possibly incomplete pig genome annotation, which suggests that the pig reference genome annotation should be improved. The target genes of these DE lncRNAs are displayed in Table 2. Next, the predicted target genes of these lncRNAs were examined using GO term and KEGG pathway analyses. A total of 1914 GO terms via *cis* and *trans* function analyses were identified in the SR vs. SS group compared to the SC group (Table S5). Among these GO terms, 115 significantly enriched GO terms (*p* < 0.01) were obtained from the *cis* and *trans* function analyses. The top 30 enriched GO terms are listed in Figure 6. For biological processes, the most enriched GO terms were related to the immune response, including the regulation of the humoral immune response, the positive regulation of T-cell-mediated immunity, the positive regulation of cytokine production and the response to bacteria. For cellular components, the main represented category was the interleukin-12 complex. For molecular functions, the main represented GO terms were interleukin-17 binding, interleukin-12 beta subunit binding and protein tyrosine kinase activity. Moreover, the KEGG pathway analysis showed that a total of 51 pathways were detected (Table S5). Among these KEGG pathways, a total of three KEGG pathways were significantly enriched (*p* < 0.05). The top 20 KEGG pathways are shown in Figure 7. Some KEGG pathways (such as inflammatory bowel disease, cytokine–cytokine receptor interaction and Leishmaniasis) were associated with the immune response and infectious diseases.

Furthermore, GO term and KEGG pathway analyses of the 89 DE mRNAs were performed. In the SR vs. SS group compared to the SC group, a total of 2336 different GO terms were obtained, and 74 GO terms were significantly enriched (corrected *p* value < 0.05) (Table S6). For biological processes, the most enriched GO terms were related to the inflammatory response, including the innate immune response and Toll-like receptor 4 binding. For cellular components, the main represented categories were extracellular space and the extracellular region. For molecular functions, the main represented GO terms were monocarboxylic acid binding and Toll-like receptor binding. A total of 10 KEGG pathways were significantly enriched (*p* < 0.05), and several immune-related KEGG pathways (such as NF-κB, Jak-STAT and TNF) were identified (Table S6). Several immune-response-related genes participated in these KEGG pathways, such as *CD14* and *DDX58* in the NF-κB signaling pathway, *HSPA1L* in antigen processing and presentation and *IL1R2* and *CD14* in the MAPK signaling pathway.

### 3.4. RT-qPCR Validation and Tissue Expression Profiling of LNC_001595

**Figure 8:**
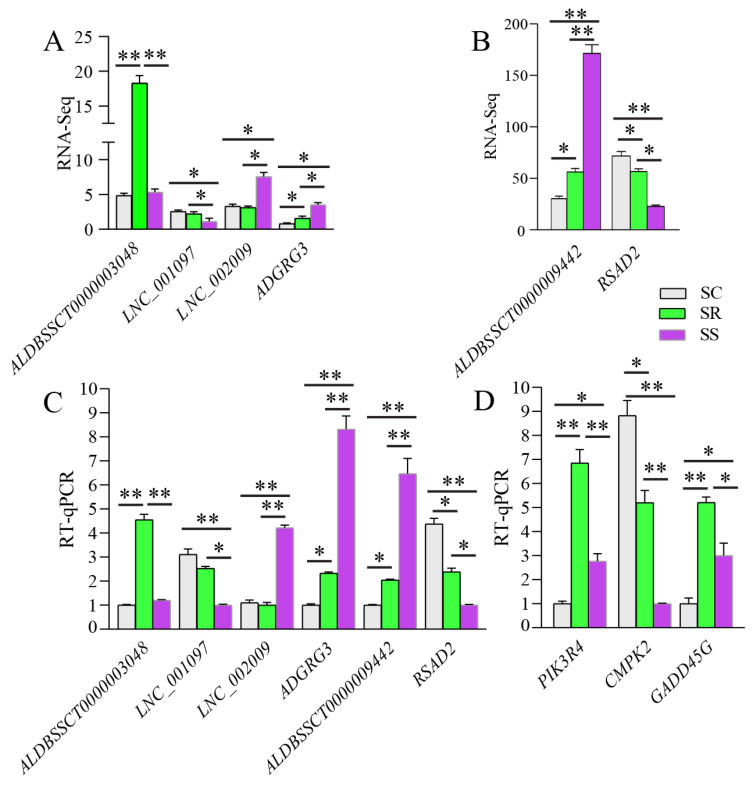
Validation of the reliability of RNA-Seq data and four gene expression levels. Data are presented as Mean ± SE with three duplicates. One-way ANOVA was performed to calculate statistical significance followed by Duncan’s test. Different asterisks above bars indicate significant differences (* *p* < 0.05, ** *p* < 0.01).

The expression levels of four DE lncRNAs and two DE mRNAs were detected using RT-qPCR to validate the reliability of our RNA-Seq data. In addition, three genes (*PIK3R4*, *CMPK2* and *GADD45G*) were selected to detect expression levels. These results show that the trends of these DE lncRNAs and mRNAs determined using RNA-Seq are consistent with those from the RT-qPCR (Figure 8A–C). Lastly, the *PIK3R4*, *CMPK2* and *GADD45G* genes were differentially expressed between the SR and SS groups (Figure 8D).

**Figure 9:**
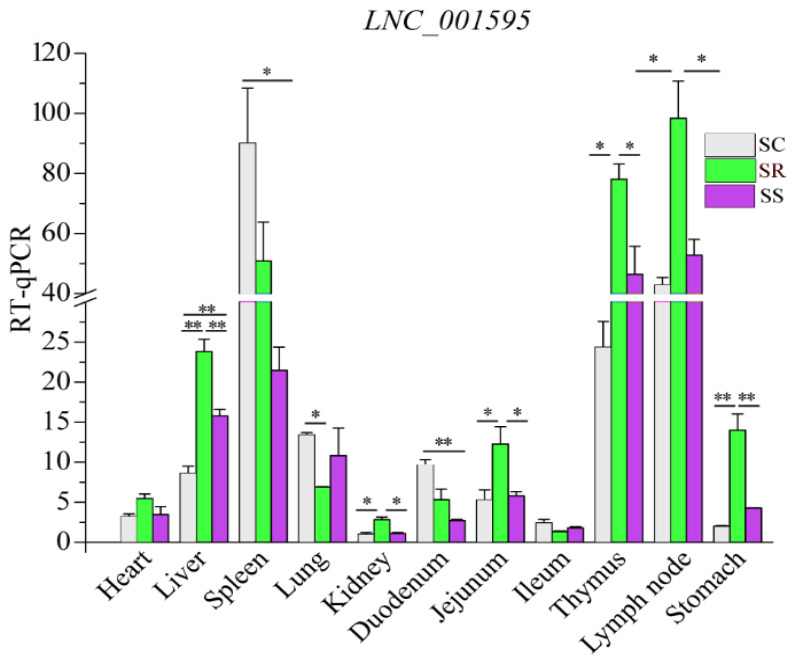
Relative expression of *LNC_001595* in different tissues. The results are shown as Mean ± SE with three duplicates. One-way ANOVA was conducted to calculate statistical significance followed by Duncan’s test. Different asterisks above bars indicate significant differences (* *p* < 0.05, ** *p* < 0.01).

Previous studies reported that one of the features of lncRNAs is their remarkable tissue specificity. Hence, to verify the tissue specificity of the lncRNAs, we selected one lncRNA (*LNC_001595*) to detect the expression level in various tissues. Expression profiling across different pig tissues indicated that the transcript *LNC_001595* was highly expressed in the spleen, lymph nodes and thymus (Figure 9).

### 3.5. The Identification of lncRNAs Antagonistic to C. perfringens Type C

**Figure 10:**
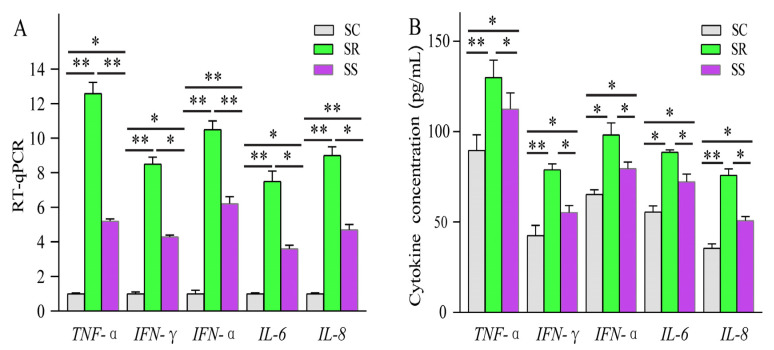
Cytokine gene expression levels (**A**) and concentrations (**B**) in the spleen. Data are displayed as Mean ± SE with three duplicates. One-way ANOVA was performed to calculate statistical significance followed by Duncan’s test. Different asterisks above bars indicate significant differences (* *p* < 0.05, ** *p* < 0.01).

**Figure 11:**
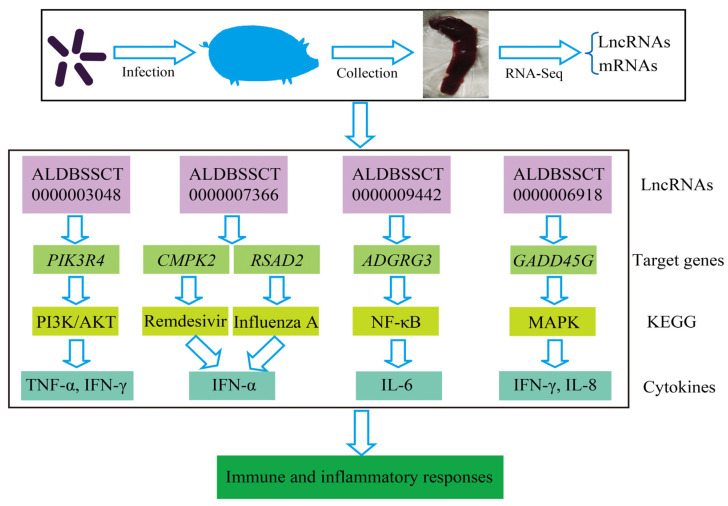
A diagram illustrating the potential lncRNAs, target genes and cytokines.

Compared with SC, a total of 14 lncRNAs and 89 mRNAs were identified as being DE in the SR vs. SS group. After *C. perfringens* type C infection, dysregulated *ALDBSSCT0000003048*, *ALDBSSCT0000009442*, *LNC_001097*, *ALDBSSCT0000007366* and *ALDBSSCT0000006918* regulated several immune genes (such as *PIK3R4*, *ADGRG3*, *RSAD*2, *CMPK2* and *GADD45G*) via *cis* and *trans*. At the same time, these DE target genes in SR vs. SS were mainly enriched in some important immune-related KEGG pathways, including inflammatory bowel disease; cytokine–cytokine receptor interaction; Toll-like receptor; and Jak-STAT, which has been reported to be related to bacterial infection, especially *C. perfringens* infection. These results suggest that the lncRNAs and their target genes had potential effects on these *C. perfringens*-related KEGG pathways. To investigate these lncRNAs’ potential roles in *C. perfringens* type C infection, two criteria were used in this study by following a previous method [37]. Firstly, differentially expressed lncRNA target genes via *cis* and *trans* were found to participate in the immune response. Secondly, the downstream immune-related cytokine genes of immune response genes were identified using KEGG pathways and some scientific research papers. Based on the above method, four lncRNAs were identified. These target genes of lncRNAs possibly indirectly or directly regulated cytokine genes in the process of *C. perfringens* type C infection. Therefore, cytokine gene expression levels and concentrations (TNF-α, IFN-γ, IFN-α, IL-6 and IL-8) were evaluated (Figure 10). To further investigate how lncRNAs function, we constructed a potential diagram among key lncRNAs, target genes and cytokines (Figure 11). For example, *ALDBSSCT0000009442* could positively regulate its target gene *ADGRG3*, and over-expressed *ADGRG3* triggered the expression of IL-6 through the NF-κB pathway to improve immunity against *C. perfringens* type C infection.

## 4. Discussion

Recently, due to the rapid development of RNA-Seq technology, lncRNAs have been regarded as new modulators related to infectious diseases, such as malaria [44], diarrhea [32], hepatitis [45] and tuberculosis [46]. Diarrhea caused by *C. perfringens* type C often leads to major economic losses in pig farms. However, the understanding of the functions of lncRNAs in the spleen of piglets infected with *C. perfringens* type C is limited. Therefore, we used deep Illumina sequencing and bioinformatics analyses to identify lncRNAs in the spleen of piglets in response to *C. perfringens* type C infection in our previous study [36]. Nevertheless, we did not comprehensively explore how lncRNAs work during *C. perfringens* type C infection. Thus, these dysregulated lncRNAs and their target genes in regulating the resistance of piglets infected with *C. perfringens* type C were investigated in this paper.

Tissue specificity is a feature of lncRNAs, unlike protein-coding genes [22]. Thus, *LNC_001595* was selected to evaluate the expression levels in different tissues. Indeed, *LNC_001595* was highly expressed in the lymph nodes and spleen, which displayed tissue specificity. Additionally, lincRNA showed a greater length, a higher expression level, a lower exon count and a shorter ORF length than those of antisense lncRNAs. These results are consistent with those of other studies [47,48].

Characterizing the structure of lncRNAs is necessary for understanding their mechanism at almost every level of gene function and regulation. The local single-chain structures, local secondary structure motifs and tertiary structure motifs of lncRNAs can interact with target genes to perform biological functions. At present, it is difficult to determine the tertiary structure of lncRNAs. The secondary structure of lncRNA target molecules is beneficial for investigating their function mechanism and exploring the relationship between the structure and function of lncRNAs. In this paper, a total of 10 lncRNAs had secondary structures, including a hairpin loop, a stem loop, an inner ring, a bulge ring and a multi-branch loop. Interestingly, the secondary structures of two lncRNAs (namely, *ALDBSSCT000000944*2 and *LNC_000263*) are consistent with two lncRNA structures in another study [37].

In order to identify the key lncRNAs against *C. perfringens* type C, we referenced two criteria by using a previous method [37]. Based on this method, a potential relationship among four lncRNAs, target genes and cytokines was identified. Four lncRNAs (namely, *ALDBSSCT0000003048*, *ALDBSSCT0000007366*, *ALDBSSCT0000009442* and *ALDBSSCT0000006918*) targeted some immune genes (such as *PIK3R4*, *ADGRG3* and *GADD45G*) via cytokines to exhibit the antagonistic effects of *C. perfringens* type C on the piglet spleen. A previous study found that the inflammation-related cytokine genes *IL-1β*, *IFN-α*, *TNF-α* and *NF-κB* in the blood had a higher over-expression after infection with *C. perfringens* type C [37]. In this study, cytokine gene expression levels and cytokine contents in the spleen were detected. Indeed, cytokine expression levels and concentrations were obviously increased in the spleen of the piglets challenged by *C. perfringens* type C. Similarly, the levels of TNF-α, IFN-γ, IFN-α, IL-6 and IL-8 were obviously increased in NE chickens caused by *C. perfringens* [15,34]. Moreover, the concentrations of these cytokines were significantly higher in those in the diarrhea group than in those in the healthy group. These results suggest that the DE lncRNAs in SR vs. SS can affect their target genes through immune-related KEGG pathways to regulate downstream cytokine genes in the piglet inflammatory and immune responses to resist *C. perfringens* type C. The higher-expressed cytokines were beneficial to the inflammation response in the piglets, which may be related to resistance to *C. perfringens* type C infection.

*PIK3R4*, also named *Vps15* or *p150*, is one of the immune genes targeted by *ALDBSSCT0000003048*, and it participates in the regulation of the autophagy pathway. *PIK3R4* as a regulatory subunit plays an important role in autophagosome formation and maturation, which is activated and accelerated under the nutrient-limiting status to supply nutrients for cell survival [49,50]. Up-regulated *PIK3R4* can enhance the process of autophagy. Additionally, *PIK3R4* plays key roles in the immune system to resist disease via the PI3K/AKT pathway [51]. In our study, the expression level of *ALDBSSCT0000003048* in the SR group was higher than that in the SS group. Correspondingly, the expression of level of *PIK3R4* in the SR group was also higher than that in the SS group. Then, up-regulated *ALDBSSCT0000003048* targeted *PIK3R4* to stimulate autophagy and secrete massive amounts of TNF-α and IFN-γ against *C. perfringens* type C infection.

*CMPK2* and *RSAD2* were regulated by *ALDBSSCT0000007366*. *CMPK2* is closely related to monocytic/macrophage terminal differentiation and played important roles in resisting pathogenic microorganisms [52,53]. *RSAD2*, also called viperin, has been reported to be associated with the innate immune response system and is commonly up-regulated by LPS, type I interferons and microorganism infections [54,55]. In our study, down-regulated *ALDBSSCT0000007366* in the SS and SR groups may have depressed the expressions of *CMPK2* and *RSAD2*. *CMPK2* via the Remdesivir pathway and *RSAD2* via the Influenza A pathway produce IFN-α for resisting *C. perfringens* type C infection.

*ADGRG3* was regulated by *ALDBSSCT0000009442.* GPR97, encoded by *ADGRG3*, is expressed in immune cells (including neutrophils, mast cells and macrophagocytes), and it is involved in many diseases’ inflammation by regulating the activity of NF-κB and then stimulating cytokine production (such as IL-6) [56,57]. Additionally, WT mice have more severe inflammation caused by obesity than ADGRG3^−/−^ mice [56]. In this study, the expression of *ADGRG3* was significantly increased after the piglets were challenged by *C. perfringens* type C, and the expression of this gene in the SS group was higher than that in the SR group. Therefore, the higher-expressed *ALDBSSCT0000009442* targeting *ADGRG3* might have some association with the expression of IL-6, and the higher-expressed IL-6 reflected that the piglets in the SR group may have immense inflammatory responses that may be beneficial to resisting *C. perfringens* type C infection.

*GADD45G* was regulated by *ALDBSSCT0000006918*. *GADD45G* plays an important role in the activation of p38 MAP kinase, which promotes IFN-γ cytokine production against pathogenic bacteria [58,59]. Indeed, the deletion of *GADD45G* genes in mice leads to an obviously reduced number of TH1 cells for resisting *Listeria monocytogenes* [60]. Compared with the SC group, the *GADD45G* expression level in the SS and SR groups was drastically increased after the piglets were infected with *C. perfringens* type C. *GADD45G* was significantly up-regulated in the SR group compared with that in the SS group. In our previous study, the expression level of *ALDBSSCT0000006918* in the SS and SR groups was obviously increased [36]. This shows that up-regulated *ALDBSSCT0000006918* targets *GADD45G* to secrete massive amounts of IFN-γ and IL-8 for resisting *C. perfringens* type C infection.

The above results indicate the relationship between the expressions of lncRNAs and their target genes, suggesting that dysregulated lncRNAs affected their target genes, causing them to participate in the biological process. Thus, these results reflect the resistance of piglets during *C. perfringens* type C infection.

## 5. Conclusions

In this study, the identification of lncRNAs in the spleen of piglets who were antagonistic or sensitive to diarrhea caused by *C. perfringens* type C was carried out. Our study indicates that DE lncRNAs regulated target genes to adjust the immune system of piglet spleen, which further influenced the difference in the piglet’s resistance to *C. perfringens* type C infection. This study provides new insights into comprehensively understanding the regulation of lncRNAs in piglet disease resistance.

## Figures and Tables

**Table 1:** Detailed information of 14 DE lncRNAs between SR and SS groups compared to SC group.

Transcript_Id	Length	Gene_Id	SR	SS	*p*	*Q*
*LNC_001595*	6577	*XLOC_093069*	1.36	0.67	0.0009	0.0196
*ALDBSSCT0000003048*	519	*ALDBSSCG0000001826*	18.27	5.33	0.0001	0.0023
*LNC_000191*	2965	*XLOC_011580*	2.11	3.81	0.0030	0.0469
*ALDBSSCT0000009442*	233	*ALDBSSCG0000005758*	56.02	171.52	0.0004	0.0114
*LNC_001065*	2890	*XLOC_064212*	0.33	1.06	0.0028	0.0449
*LNC_001987*	20,029	*XLOC_118880*	2.89	4.73	0.0010	0.0220
*LNC_000042*	4774	*XLOC_002451*	1.00	0.45	0.0027	0.0438
*ALDBSSCT0000006918*	1302	*ALDBSSCG0000004208*	14.20	33.32	0.0001	0.0041
*LNC_001097*	8758	*XLOC_065996*	2.21	1.13	0.0008	0.0181
*LNC_000263*	280	*XLOC_016001*	12.34	23.03	0.0001	0.0023
*LNC_002009*	1490	*XLOC_120225*	3.13	7.56	0.0001	0.0041
*ALDBSSCT0000007366*	504	*ALDBSSCG0000004471*	27.97	13.09	0.0002	0.0056
*LNC_001253*	13,487	*XLOC_074796*	0.59	1.53	0.0001	0.0023
*LNC_001985*	31,206	*XLOC_118644*	2.25	1.45	0.0030	0.0469

**Table 2:** Target gene prediction of potential diarrheic resistance lncRNAs.

LncRNA Transcript_Id	*Trans* Target Genes	*Cis* Target Genes
*ALDBSSCT0000003048*		***PIK3R4***
*LNC_000191*	*GTPBP8*	
*ALDBSSCT0000009442*	***ADGRG3***	*SPOCD1*
*LNC_001987*	*ZNF648*	
*ALDBSSCT0000006918*	*FAM167B, **IL17RD**, GADD45G, LY6G5B, ZGRF1, IQCJ, LRFN5, DDR1, NCALD, **IL17F**, POU4F3, **IL17RA**, SLC2A13, CCDC110, LIN28A, DEFB123, MUSK, **CD63**, TTYH1, RXRB, RBFOX1, MCAT, GGT1, CA7, SYCN, MRC2, CALCRL, PDZD4, HIST1H2BA, MROH1, SPINK5, **IL12A**, PGPEP1L, PAG6, PNCK, ICAM2, MARCH3, ADAMTS4, SSMEM1, PLAC8L1, LCN8, HRASLS5, SLC39A14, ONECUT2*	
*LNC_001097*		*TFEC*
*LNC_000263*	*RPL8, SPO11, MAEL, BTG4, WEE2, NLRP9, OTX2, H1FOO, HAL, FKBP6, PAX6, FOXR1, TCL1A, BCL2L10, ZP4, HMGB2, NLRP11*	*ZC3H7A*
*ALDBSSCT0000007366*		***RSAD2**, RNF144A, **CMPK2***
*LNC_001985*		*ZNF398, PDIA4, ZNF425, ZNF786*

Note: the overstriking and italic letters represented as genes are related to infectious diseases, immune diseases and inflammation.

## Data Availability

The sequencing data were submitted to the Genome Expression Omnibus (Accession numbers GSE105797) in NCBI.

## Supplementary Materials

The following supporting information can be downloaded at https://www.mdpi.com/article/10.3390/cimb45030149/s1, Table S1: Primer sequence for mRNAs, lncRNAs and cytokine genes; Table S2: Differentially expressed lncRNAs and mRNAs between SS and SC; Table S3: Differentially expressed lncRNAs and mRNAs between SR and SC; Table S4: Target genes of 14 lncRNAs in *cis* and *trans*; Table S5: GO enrichment and KEGG pathway analyses of the protein-coding target genes of 14 lncRNAs; Table S6: Functional enrichment analysis of 89 differentially expressed mRNAs.
